# Assessing the Measurement Precision of Various Arsenic Forms and Arsenic Exposure in the National Human Exposure Assessment Survey (NHEXAS)

**DOI:** 10.1289/ehp.8104

**Published:** 2005-10-13

**Authors:** Edo D. Pellizzari, C. Andrew Clayton

**Affiliations:** RTI International, Research Triangle Park, North Carolina, USA

**Keywords:** arsenic species, children, drinking water, food, human exposure, National Human Exposure Assessment Survey, NHEXAS, population study, urine

## Abstract

Archived samples collected from 1995 to 1997 in the National Human Exposure Assessment Survey (NHEXAS) in U.S. Environmental Protection Agency Region 5 (R5) and the Children’s Study (CS) in Minnesota were analyzed for total arsenic, arsenate [As(V)], arsenite, dimethyl arsenic acid (DMA), monomethyl arsenic acid (MMA), arsenobetaine (AsB), and arsenocholine. Samples for the CS included drinking water, urine, hair, and dust; both studies included food (duplicate plate, composited 4-day food samples from participants). Except for AsB and As(V), the levels for As species measured in the food and drinking water samples were very low or nonexistent. The analytical methods used for measuring As species were sensitive to < 1 ppb. During the analysis of food and drinking water samples, chromatographic peaks appeared that contained As, but they did not correspond to those being quantified. Thus, in some samples, the sum of the individual As species levels was less than the total As level measured because the unknown forms of As were not quantified. On the other hand, total As was detectable in almost all samples (>90%) except for hair (47%), indicating that the analytical method was sufficiently sensitive. Population distributions of As concentrations measured in drinking water, food (duplicate plate), dust, urine, and hair were estimated. Exposures to total As in food for children in the CS were about twice as high as in the general R5 population (medians of 17.5 ppb and 7.72 ppb, respectively). In addition, AsB was the most frequently detected form of As in food eaten by the participants, while As(V) was only rarely detected. Thus, the predominant dietary exposure was from an organic form of As. The major form of As in drinking water was As(V). Spearman (rank) correlations and Pearson (log-concentration scale) correlations between the biomarkers (urine, hair) and the other measures (food, drinking water, dust) and urine versus hair were performed. In the NHEXAS CS, total As and AsB in the food eaten were significantly correlated with their levels in urine. Also, levels of As(V) in drinking water correlated with DMA and MMA in urine. Arsenic levels in dust did not show a relationship with urine or hair levels, and no relationship was observed for food, drinking water, and dust with hair. Urine samples were collected on days 3, 5, and 7 of participants’ monitoring periods. Total As levels in urine were significantly associated across the three pairwise combinations—i.e., day 3 versus day 5, day 3 versus day 7, and day 5 versus day 7. Because the half-life of As in the body is approximately 3 days, this suggests that some exposure occurred continually from day to day. This trend was also observed for AsB, suggesting that food is primarily responsible for the continual exposure. DMA and MMA in urine were also significantly correlated but not in all combinations.

The adverse health effects of exposure to high arsenic levels, including a deterioration of skin on the hands (Dibner 1958), were recognized as early as 1556. The effects of exposure to As were reported four centuries later by Hutchison, who described skin carcinoma in patients treated with arsenical-based compounds (Hunter 1957).

Subsequently, inhalation of inorganic As was found to produce lung cancer [International Agency for Research on Cancer (IARC) 1980], and studies in the 20th century have shown increased risks of skin, liver, lung, bladder, and kidney cancers in Taiwanese, Mexican, Indian, German, Argentinean, and Chilean populations [Agency for Toxic Substances and Disease Registry (ATSDR) 1989; Bergoglio 1964; Biagini et al. 1978; Cebrian et al. 1983; Chakraborty and Saha 1987; Chen et al. 1985, 1986, 1988a, 1994; Chen and Wang 1990; Chiang et al. 1988; Dang et al. 1983; U.S. Environmental Protection Agency (EPA) 1988; Science Applications International Corporation (SAIC) 1987; Tseng et al. 1968; Tsuda et al. 1990; Wu et al. 1989; Yamauchi and Yamamura 1983; Zaldivar 1974; Zaldivar et al. 1981] and skin lesions in Bangladesh subjects (Rahman and Axelson 2001; Yu et al. 2003) who ingested As-contaminated drinking water.

The occurrence of total As in drinking water and in food has been reported (Branch et al. 1994; Hwang and Jiang 1994; SAIC 1987; Thomas and Sniatecki 1995). Both organic and inorganic forms of As are present in varying amounts. Fish and shellfish contain relatively high concentrations of total As, with levels reaching into the parts per million range. However, most of the As is in the organic form as arsenobetaine (AsB) (Velez et al. 1995). Drinking water surveys have reported that most major supplies contain < 5 ppb of total As, but levels > 50 ppb do occur in some areas of the United States (SAIC 1987). Inorganic As can be present in drinking water as either arsenate [As(V)] or arsenite [As(III)].

Total As has been reported in soil and house dust at 0.2–40 ppm and 0.2–400 ppm, respectively (Fernando et al., unpublished data). Because urban air levels for As occur at about 20 ng/m^3^, inhalation is not considered a significant route of environmental exposure (IARC 1980).

Of the three possible routes of exposure (inhalation, ingestion, and dermal) to As, ingestion is potentially the greatest contributor to exposure, with drinking water and food the two primary ingestion pathways. However, there is a paucity of population-based exposure data that describes the total ingestion (also referred to as intake) of the different forms of As from the combination of drinking water and food. The extent of population exposure occurring from a combination of these pathways is not well understood. Understanding such relationships may improve future exposure and risk assessments for As.

Based on the current knowledge of As levels in the environment, the primary exposure to As is potentially through ingestion; however, a probability-based exposure distribution of arsenical species from ingesting drinking water and food has not been previously reported for the population in the Great Lakes (USA) area. Both pathways are the focus of this study. In this article we report the contribution of total As and its species from dietary sources to exposure of a general population and in children from the National Human Exposure Assessment Survey (NHEXAS) conducted in U.S. EPA Region 5 (R5) (Pellizzari et al. 1995).

## Methods

### Study design and populations for collected U.S. EPA Region 5 and Children’s Study samples.

The NHEXAS conducted in R5 and the Minnesota Children’s Pesticide Exposure Study (CS), a module of the NHEXAS that focused on children 3–12 years of age, are probability-based surveys of noninstitutionalized persons that provided multimedia environmental concentration data, exposure data, and biomarker data. The R5 study was conducted in 1995–1996 and involved the monitoring of approximately 250 participants residing in the six states surrounding the Great Lakes. The CS, conducted in the summer of 1997, involved similar monitoring for 102 children living in Minneapolis/St. Paul, Minnesota, and in two rural Minnesota counties. These NHEXAS studies have been described in previous papers, including papers on design and measurement issues (Pellizzari et al. 1995; Quackenboss et al. 2000) and on survey design, weighting, and response rates (Whitmore et al. 1999).

### Samples collected for arsenic analysis.

Table 1 lists the samples available from the studies and the data derived from these samples for total As and its forms.

#### Food samples.

Four-day composite food samples collected from 1995 to 1997 in R5 and in 1997 in the CS were extracted and analyzed for total and As species (Table 1). Sample collection methods have been previously described (Pellizzari et al. 1995; Thomas et al. 1999). The samples were collected, homogenized, and stored in 50-mL polypropylene tubes at −20°C until analysis.

#### Drinking water sample.

Drinking water samples collected from 102 homes in the CS were available for measuring total and speciated As (Table 1). Sample collection methods have been described elsewhere (Thomas et al. 1999). Briefly, the samples were collected in 50-mL polypropylene tubes and stored at −20°C. As part of the quality control (QC) assessment, field controls (FCs) were prepared in the laboratory by spiking As(V), As(III), dimethyl arsenic acid (DMA), and monomethyl arsenic acid (MMA) in deionized water at 50 ng/mL. They were taken to the field, kept with the samples, and stored frozen along with the samples. Laboratory controls (LCs), which were prepared and stored frozen but not taken to the field, were intended to show that As species were preserved by freezing over time (samples were collected in 1997 and analyzed in 1999).

#### Urine samples.

Urine samples collected from subjects on days 3, 5, and 7 of the monitoring period in the CS in 1997 were made available for measuring total and speciated As (Table 1). The samples were collected and stored in 50-mL polypropylene tubes at −20°C until analysis in 2000 (Pellizzari et al. 1995; Quackenboss et al. 2000).

All food, water, and urine samples with total As levels below the detection limit were not analyzed for individual As species. For these cases, a zero value was imputed for statistical analysis.

#### House dust and hair samples.

House dust and hair samples collected in the CS were available for measuring total As levels. The samples had been stored in polypropylene bags at −20°C until analysis in 2000 (Pellizzari et al. 1995).

### Sample analysis.

Drinking water, food, and urine samples were analyzed for total and As species using previously reported methods (Milstein et al. 2002, 2003a, 2003b). At the beginning of sample analysis, an eight-point calibration curve was prepared covering the range from 0.05 to 50 ppb As. Every batch of samples analyzed included a calibration check (1 and 10 ppb), a calibration blank (0 ppb), 10 field samples, a control sample [a standard reference material (SRM) or LC for drinking water, and a method control (MC) or certified reference material (CRM) for food and hair], and an independent check standard (10 ppb). The calibration check standard was used to assess sensitivity as judged by the total area counts for As and the bias of the calibration curve prior to the analysis of samples. The calibration blank served to assess any background carryover in the ion chromatographic system. The independent check standard at the end of the batch of samples was used to assess ion chromatograph inductively coupled plasma-mass spectrometer (ICP-MS) stability or drift from the original calibration curve. In addition, duplicate samples (DS) were analyzed to assess reproducibility. Table 2 summarizes the types of QC samples used.

### Available arsenic data.

For both R5 and the CS, the As food measurements were for composite (duplicate diet) samples of solid foods consumed over a 4-day period (days 4–7 of a participant’s monitoring period); both As concentrations (micrograms per kilogram) and intakes (micrograms per day) were determined for the food samples. We calculated intakes from the amount of food consumed per day times the concentration in the food composite.

For the CS, As data were also available from three urine samples (nanograms per milliliter) obtained on days 3, 5, and 7 of the participant’s monitoring period, from drinking water, and from house dust and hair samples (total As only). The basic unit of observation that represents the integrated exposure period measured is a person-period for the food (4 days), urine (first morning void), and hair (1.5 months) data, and a household-period for the drinking water and dust data. Thus, distributional estimates determined for these various media are for distributions over those respective units.

### Statistical methods for analysis of quality control data.

We computed summary statistics for the blank, control, and DS and duplicate analyses. Analytical bias was assessed by determining the amount of background contribution in blanks and by the percent As recovered in control samples, i.e., a comparison of the measured to a certified or known amount. This was quantitatively judged by the mean recoveries and coefficient of variation (CV) for paired observations.

We first assessed analytical precision by calculating standard deviations (SDs) and relative standard deviations (RSDs) of the duplicate analyses; similar measures were determined for the replicate aliquots. We computed these statistics when both observations of a pair had measurable values above the detection limit. The duplicate extract/digest analysis SDs and RSDs include only the instrumental analytical error, whereas replicate-aliquot measures include variability associated with preparation of aliquots, extraction in the case of food, as well as the instrumental analysis. The various aspects of precision were judged by summarizing the distributions of the SDs and RSDs over various cases. The sample size, the minimum, median, mean, and maximum were determined.

### Statistical methods for analysis for field samples.

Proper analysis of data collected for members of a probability sample requires that all observations be weighted inversely to their probabilities of selection. These sampling weights enable design-unbiased estimation of linear population parameters such as population totals. Initial sampling weights were developed as a part of the sample design activities of the R5 and CS; after data collection, these sampling weights are adjusted to compensate (at least partially) for the potential bias resulting from survey nonresponse. We used weighting class adjustment procedures in those studies to make the adjustments. The paragraphs below indicate how the adjusted sampling weights were employed in making estimates of various population parameters.

A common example requiring weighted data analysis is the estimation of a population proportion. For instance, for estimating a proportion *P_x_*, the general form of the estimate is


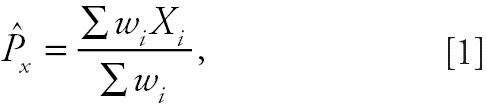


where the summations are over all sample participants, *w_i_* denotes the sampling weight associated with participant-period (or house-hold-period) *i*, and *X_i_* is an indicator variable with a value of 1 if participant-period *i* has the characteristic of interest and with a value of 0 otherwise. The numerator is an estimate of the total number of participant-periods (or household-periods) in the population having the characteristic, and the denominator is an estimate of the total number of participant-periods (or household-periods) in the population. This type of estimate is used, for instance, to produce a weighted estimate of the percent measurable (e.g., the estimated percent of the population of person-periods with detectable levels of a given As species) by setting *X* = 1 for all observations with a detectable level, and setting *X* = 0 for all nondetects.

If *Y_i_* denotes a continuously measured quantity for observation *i* (e.g., the As total concentration in food), then a similar expression is used to estimate the mean of the target population:


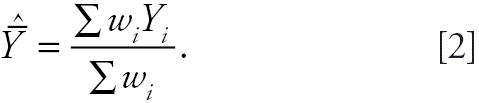


The numerator estimates the total of the *Y* variable that would have been obtained if all members of the target population had been observed; as before, the denominator estimates the total size of the target population.

In addition to estimating such population parameters (e.g., proportions, means), it is important to estimate the precision of the estimate, which is usually expressed in terms of its variance or standard error. The estimation of sampling variances and standard errors for statistics calculated from probability sampling data should be based on the randomization distribution induced by the sampling design (i.e., they should account for all features of the sampling design, such as stratification and multistage sampling). Such an approach is robust because it makes no assumptions regarding the distribution of occurrence (e.g., normality) of the survey items. Hence, analyses based on the design-induced distribution provide the most defensible basis for making inferences from the sample to the target population.

The classic approach to estimating standard errors for nonlinear statistics such as means and proportions from complex probability sampling designs is a first-order Taylor Series linearization method, which was the method employed in this study. Alternative variance estimation techniques for such designs include jackknifing and balanced repeated replication. RTI used its special purpose survey data analysis (SUDAAN) software to analyze complex survey data (RTI International, Research Triangle Park, NC). SUDAAN estimated the standard errors using the classical Taylor Series method because such estimates are both computationally and statistically efficient. This software includes procedures for survey based estimation of standard errors of population totals, means, proportions, and ratios, as well as linear and logistic regression relationships. For means, proportions, differences in means, or differences in proportions, the precision is generally reported as an approximate 95% confidence interval calculated as the estimate ± two times the standard error of the estimate.

The method for calculating measures of precision for percentiles was somewhat different. First, the percentile estimate (e.g., for the *p*th percentile) was determined by forming a weighted cumulative empirical distribution and determining the point (e.g., *X_p_*) at which the sum of the weights was 100*_p_*% of the total sum of the weights. A domain consisting of all observations with observed values less than *X_p_* was then formed, and the proportion of the population falling into this domain (approximately equal to *p*) was estimated as *p̂*. The standard error of this estimate was formed via the Taylor’s Series method, and a confidence interval for *p* was formed as [*p̂* − *t_a_SE*(*p̂*) + *t_a_SE*(*p̂*)], where *t_a_* is an appropriate tabulated *t* value. An inverse interpolation of the empirical cumulative distribution was then used to translate this interval into one for the percentile. That is, the lower confidence limit was that point *L_p_* at which 100[(*p̂* − *t_a_SE* *p̂*)] % of the total sum of the weights occurs, and the upper confidence limit was that point *U_p_* at which 100[(*p̂* − *taSE* *p̂*)] % of the total sum of the weights occurs. This interval, [*L_p_*, *U_p_*], forms an interval estimate for the *p*th percentile; it is typically asymmetric about *X_p_*. The interval was translated into a standard error by dividing the interval length (*U_p_–L_p_*) by *2t_a_*. Although such a standard error statistic cannot be used along with the estimated percentile to directly construct a confidence interval, it can be used to indicate the precision of one estimated percentile relative to another.

Because some media and chemicals exhibited a low percent measurable, the above types of weighted summary statistics (e.g., means and percentiles) and associated confidence intervals were generated only for those media/chemicals with ≥ 10% measurable; those weighted statistics employed half the detection limit for all nondetects.

In addition to the weighted statistics, we generated various Spearman (rank) correlations and weighted Pearson correlations; the latter were performed for logarithms of the concentrations, because the log-scaled data tended to be more symmetrically (and normally) distributed.

## Results and Discussion

### Quality control data.

The QC results for the calibration blanks indicated that the background was less than the lowest calibration standard (0.05 ppb) for all days of analysis for As species. The bias between the nominal As level in the standard and that calculated was determined for each As form in each batch of samples analyzed. In most cases, this bias was < 10%. Percent recovery was used to evaluate how well the instrumental analysis system performed on the check standards. The percent recovery for the 1-ppb and 10-ppb check standards were generally excellent, ranging from 86 to 107.

The results for total As measurement in field blanks indicated that no major contamination was associated with the vessels used to collect, store, and process the food, drinking water, hair, and dust samples. These results for total As were also applicable to As speciation.

We used CRM (food, hair), MCs (food), LCs and FCs, and SRMs (drinking water) to assess bias of the analysis methods. The results for these samples were expressed as a percent recovery (ratio of measured to known values). A summary is given in Table 3, which provides the number of QC samples of each type, the mean of the percent recoveries, and the CV of the percent recoveries. The percent recoveries were excellent in most cases for each of the As forms across the media.

We used duplicate injection (DI) of the same sample extract, duplicate analysis of an aliquot of the same sample (DA), and analysis of DS to assess precision of the instrumental method, the analysis method, and the overall collection and analysis methods, respectively, for selected As forms. Percent RSDs were determined for each pair, and the distributions of these RSDs were then summarized in terms of a mean, median, and maximum. These results for DI and DA pairs are given in Table 4 for food, drinking water, and urine. Except for drinking water, the DI and DA median percent RSDs were < 26%. For As(V) in drinking water, one pair had a large SD, but the reason for this could not be determined.

Table 5 presents the results of analysis for DS for dust and drinking water. In general, the precision associated with processing and extracting As of the sample was less than the precision for DA.

### NHEXAS field data.

Table 6 lists the number of samples speciated for As and the number of samples in which total As was measured. It also provides statistically weighted estimates of the percentage of samples with measurable values above the detection limits. These percentages represent estimates of those expected if the entire target populations were subjected to the data collection and analysis methodologies used in the R5 and the CS. The analytical methods used for measuring As species were sensitive to < 1 ppb.

The highest percent measurable values occurred for total As across all samples (> 90%, Table 6). This was expected because the detection limit was lower for total As than for any of the forms measured. AsB had the highest percent of measurable values in food. In a few samples, As(V) was also detected in food.

The most prevalent As form in water was As(V), whereas As(III) was measurable in a few samples. This compared with DMA in urine, which was measurable in up to 73% of the samples. Arsenocholine (AsC) was essentially not found any of the samples. In food, the most prevalent form was AsB.

During the analysis of food and drinking water samples by ion chromatograph ICP-MS, chromatographic peaks appeared that contained As, but they did not correspond to those being quantified. Thus, in some samples, the sum of the individual As species levels was less than the total As level measured, because the unknown forms of As were not quantified. In addition to the measured As forms reported here, there are as many as 18 other forms that have been identified in environmental and biological systems (Francesconi et al. 1999; Le et al. 1999, 2004; Miguens-Rodriguez et al. 2002; Montilla et al., unpublished data; Sanchez-Rodas et al. 2002; Schmeisser et al. 2004; Soeroes et al. 2005). These forms include dimethylarsinoylethanol, several arsenosugars, and thioarsenosugars found in shrimp, oysters, and seaweed.

Table 7 furnishes estimates of the population distributions for selected media and As species (or total). Inestimable percentiles (shown as “—” in the table) occur for some of the lower percentiles because of the degree of nondetects that occur.

Table 7 also provides the approximate 95% confidence interval estimates for the parameters. Inestimable cases (shown as blanks in the table) sometimes occur for lower percentiles because there is no variability among the nondetects; they sometimes occur for upper percentiles because the empirical distribution is too flat to allow inverse interpolation to be carried out.

It is evident from the distributional results (Table 7) that the exposure of children to total As in food was about twice as high as that of the general R5 population (e.g., medians of 17.5 ppb and 7.72 ppb for the CS and R5, respectively). However, as indicated in Table 6, AsB was the most frequently detected As form in food eaten by the participants, while As(V) was only very rarely detected.

For selected As forms, Table 8 shows Spearman (rank) correlations between the biomarkers (urine, hair) and the other measures (food, drinking water, dust); it also shows urine versus hair correlations. In the CS, total As and AsB in the food eaten was significantly correlated with their levels in urine (Table 8). In addition, levels of As(V) in drinking water correlated with DMA and MMA in urine (day 3). More statistically significant Pearson (log-scale) correlations of total As and its species (Table 9) were found than were found via the Spearman method, but the general trend of food and urine relationships were similar (Table 9). Total As levels in dust did not show a relationship with urine or hair. We observed no relationships for food, drinking water, or dust with hair.

Urine samples, as previously noted, were collected on days 3, 5, and 7 of participants’ monitoring periods. Correlations among these data are presented in Table 10. Total As levels in urine were significantly associated across the three pairwise combinations, for example, day 3 versus day 5. Because the half-life of As in the body is approximately 3 days, this suggests that some exposure continually occurred from day to day. This trend was also observed for AsB, which suggests that food is responsible for the continual exposure. DMA and MMA in urine were also significantly correlated but not in all combinations.

The combination of ingestion and metabolism of inorganic and organic As yields a complex array of As forms in human urine (Aposhian et al. 2004; Donohue and Abernathy 2001; Hansen et al. 2004; Styblo et al. 2001; Thomas et al. 2001; Vahter 1999), which accounts for the combination of correlations observed between the various As forms ingested and DMA and MMA in urine from NHEXAS subjects. Most studies indicate, on average, 10–30% inorganic As, 10–20% MMA, and 60–70% DMA in urine, but the methylation of As is governed by its absorption, dose level, route of exposure, and age (Vahter 1999). The relative levels measured in urine for NHEXAS (Tables 6 and 7) are consistent with these reported observations.

## Summary

### Data quality.

Before interpreting results derived in this study, the QC data from chemical analyses were thoroughly analyzed to establish the level of quality that was achieved. In general, data quality was considered excellent, very good, or acceptable if the precision or bias was < 10%, < 20%, or < 30%, respectively. A summary for each facet of the study follows.

### Drinking water sample analysis.

#### Total arsenic.

The QC results derived from calibration blanks indicated that the background was less than the lowest calibration standard (0.05 ppb) for all days of analysis. The bias between the As level in the 1-ppb calibration standard and that determined from a standard curve was in most cases < 10%. Percent recovery for 1-ppb and 10-ppb check standards was used to evaluate how well the instrumental analysis system performed. In general it was very good, ranging from 86 to 107%.

The inclusion of SRMs during the analysis of water samples permitted the assessment of precision and bias. The precision was ≤ 4% across all batches of samples analyzed, and the bias was ≤ 6%.

Field blanks were included in the NHEXAS study, and results indicated that no major contamination was associated with the vessels used to collect, store, and process the samples. These results for total As were also applicable to As speciation.

Drinking water controls containing known amounts of As were included in the NHEXAS study, and the percent recoveries were excellent. DA and the analysis of DS permitted an assessment of precision of the analysis method and the collection and analysis methods, respectively. The percent RSD across duplicate pairs was excellent. DA was also performed, with very good results.

#### Arsenic species.

Except for the first analysis batch, the measurement bias, in general, was ≤ 10%. Overall, the results were judged very good. The percent recoveries for LCs and FCs were determined for As(V), As(III), DMA, and MMA. For the LCs, they were very good to excellent, ranging from 97% to 122% across the four As species. The FCs were excellent, ranging from 100% to 105%. No field blanks were included for QC purposes, because total As measurements indicated the blanks contained little background and it was below the detection limit for the speciation method.

The precision of the instrumental method was assessed by performing DIs of the same sample for As(V). The mean percent RSDs were very large because one pair had a large standard deviation. The reason for this could not be found. Sufficient pairs (five) of DS with measurable values of As were found only for As(V). The mean percent RSD across the duplicate pairs was 26%, which was considerably better than for DIs. Based on these results, the data were deemed acceptable.

### Results for urine sample analysis.

#### Total arsenic.

Except for a few cases, the percent bias for total As quantification was ≤ 10% across the calibration standards and QC check standards. In cases where the bias was large, the analysis of the set of samples was repeated. The calibration blank contained negligible traces of As.

Duplicate analysis of sample extracts for total As permitted an evaluation of instrumental precision. The instrumental precision was excellent (mean percent RSD < 10%). The results for individual DS pairs, a measure of method precision, were very good (mean percent RSD 13%).

#### Arsenic species.

The RSD between the initial calibration standard and the QC check standard was ≤ 30% across the six As species and in many cases was < 10%. In cases where measurable values for As species were observed in both DS, reproducibility, as expressed as the RSD for each pair, was acceptable.

A summary of the results for paired RSDs across the few DIs and samples available with measurable values for As species found in the urine and the observed precision for the method yielded acceptable results.

### Results for food sample analysis.

#### Total arsenic.

No As was detected in the blanks. Thus, these blanks were not included in the speciation analysis. The bias, expressed as percent recovery, was estimated using a CRM and MC samples. The mean recovery was excellent (100%) for both QC samples. However, the recoveries were very good to acceptable with the ranges for CRM and MC samples (66 to 141% and 90 to 112%, respectively).

#### Arsenic species.

Calibration check sample results were used to assess stability of the instrument calibration. The precision expressed as percent RSD was generally very good for the six species (< 20%).

From DA, results were available only for AsB. These results were used to assess instrumental precision of analysis. For four analysis pairs, the precision was very good. DS results permitted a measure of the precision of composite food aliquoting and method of analysis. As expected, the instrumental precision was better than the method precision. The variability was due partly to the variation in AsB between samples, i.e., at lower levels, the percent RSD was larger. The mean percent RSD for AsB was 10% and 30% for instrumental and method analyses, respectively.

### NHEXAS field samples.

Raw data from the analysis of As in drinking water, hair, dust, food [duplicate plate, composited 4-day food samples (days 4–7) from the participants], and urine (days 3, 5, and 7) were available for statistical evaluation. Except for AsB and As(V), the levels for As species measured in the samples were very low or nonexistent in food and drinking water. (The analytical methods used for measuring As species were sensitive to < 1 ppb.) During the analysis of food and drinking water samples, chromatographic peaks appeared that indicated As, but these did not correspond to the As species being quantified. Thus, in several samples there was underreporting of As species concentrations, because some forms of As were not quantified. On the other hand, total As was detectable in almost all samples (> 90%) except for hair (47%), indicating that the analytical method was sufficiently sensitive.

**Figure 1:**
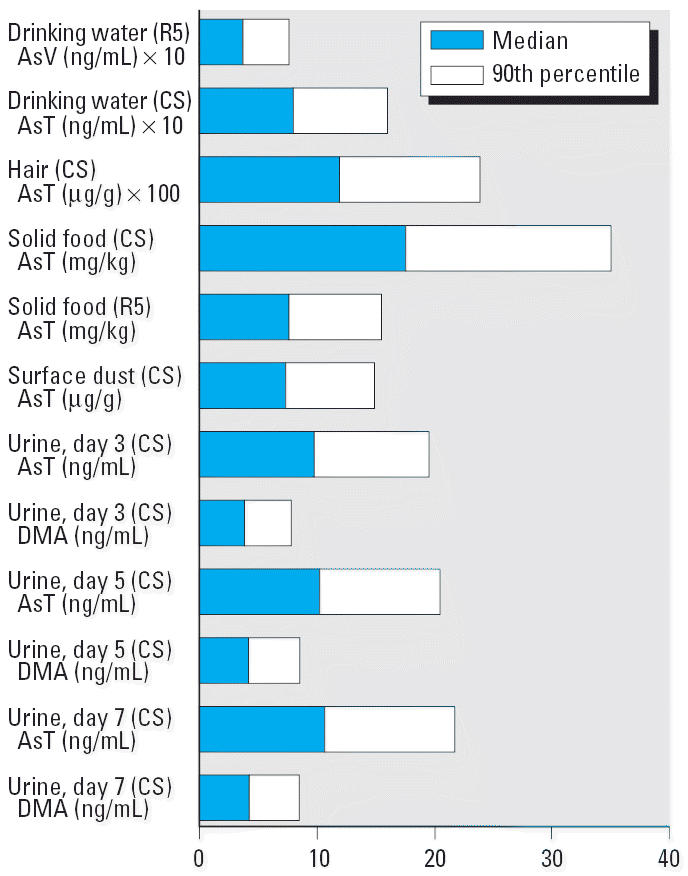
Distribution of arsenic species in environmental and human biological samples. AsT, total As.

It was evident from the distributional results (Figure 1) that the exposure of children to total As in food was about twice as high as the general R5 population (e.g., medians of 17.5 and 7.72 ppb for the CS and R5, respectively). AsB was the most frequently detected species in food eaten by the participants, whereas the more toxic As(V) was only rarely detected (i.e., the predominant dietary exposure was from an organic form of As.)

Both Pearson (log-scale) and Spearman (rank) correlations between the biomarkers (urine, hair) and the other measures (food, drinking water, dust) and urine versus hair were performed. In the CS, total As and AsB in food were significantly correlated with their levels in urine. Levels of As(V) in drinking water exhibited significant correlations with DMA and MMA in urine. Arsenic levels in dust did not show relationships with urine or hair. We observed no relationships for food, drinking water, and dust with hair.

The major findings of the study included *a*) acceptable to excellent data quality in As exposure and biomarker measurements; *b*) confirmation of the presence of the As species expected in water [(As(V)], in food (AsB), and in urine (MMA and DMA); *c*) some significant associations between exposure and biomarker levels of As and its species; and *d*) the low level of personal exposure to toxic forms of As in R5. The lack of some other associations is likely due to the various times of measurement and the transformations and half-lives that As species undergo within the body.

## Figures and Tables

**Table 1:** Samples and available data.

			As forms						
Study	Media	No.^a^	Total	As(V)	As(III)	DMA	MMA	AsB	AsC
R-5	Food	159	Y	Y	Y	Y	Y	Y	Y
CS	Food	99	Y	Y	Y	Y	Y	Y	Y
	Water	102	Y	Y	Y	Y	Y	N	N
	Urine, day 3	79	Y	Y	Y	Y	Y	Y	Y
	Urine, day 5	83	Y	Y	Y	Y	Y	Y	Y
	Urine, day 7	83	Y	Y	Y	Y	Y	Y	Y
	Dust	101	Y	N	N	N	N	N	N
	Hair	79	Y	N	N	N	N	N	N

Abbreviations: N, no; Y, yes.

a Number of observations.

**Table 2:** QC samples available.

		As form						
Sample type	Media	Total	As(V)	As(III)	DMA	MMA	AsB	AsC
Blanks	Food	Y	N	N	N	N	N	N
	Water	Y	N	N	N	N	N	N
	Urine	Y	Y	Y	Y	Y	Y	Y
	Dust	Y	N	N	N	N	N	N
	Hair	Y	N	N	N	N	N	N
Controls	Food	Y	N	N	N	N	N	N
	Water	Y	Y	Y	Y	Y	N	N
	Hair	Y	N	N	N	N	N	N
Duplicate analysis	Food	Y	Y	Y	Y	Y	Y	Y
	Water	Y	Y	Y	Y	Y	N	N
	Urine	Y	Y	Y	Y	Y	Y	Y
Duplicate samples	Food		Y	Y	Y	Y	Y	Y
	Water	Y	Y	Y	Y	Y	N	N
	Urine	Y	Y	Y	Y	Y	Y	Y
	Dust	Y	N	N	N	N	N	N

Abbreviations: N, no; Y, yes.

**Table 3:** Percent As recoveries from control samples used in CS.

Media	Type	As form	No.^a^	Mean	CV (%)
Food	CRM	Total	8	100	34
	MC	Total	8	100	8.2
Hair	CRM	Total	11	98	12
Water	FC	As(V)	11	101	34
		As(III)	11	103	37
		DMA	11	105	14
		MMA	11	104	14
		Total	10	100	1.9
Water	LC	As(V)	5	122	17
		As(III)	5	97	21
		DMA	5	113	20
		MMA	5	116	18
		Total	10	83	33
Water	SRM	As(V)	2	66	4.5
		Total	12	103	3.1

a Number of observations.

**Table 4:** Arsenic results for duplicate analyses of food, drinking water, and urine samples in the NHEXAS.

					Percent RSD		
Study	Media	Type	As form	No.^a^	Mean	Median	Maximum
R5 and CS	Food	DA	AsB	8	30	26	104
R5 and CS	Food	DI	AsB	4	10	9.5	19
CS	Water	DI	As(V)	6	76	74	108
			Total	1	11	11	11
	Urine	DA	DMA	1	14	14	14
			MMA	1	23	23	23
			Total	18	13	9.4	34
	Urine	DI	As(V)	2	26	26	43
			As(III)	1	3.2	3.2	3.2
			DMA	16	5.8	3.6	23
			MMA	3	15	17	18
			AsB	8	9.0	3.9	25
			Total	4	6.8	6.9	11

a Number of pairs.

**Table 5:** Arsenic results for DS of surface dust and drinking water in CS.

				Percent RSD		
Media	As form	Units	No.^a^	Mean	Median	Maximum
Dust	Total	ng/cm^2^	6	32	32	64
Dust	Total	μg/g	6	14	5.1	36
Water	As(V)	ng/mL	5	26	28	42
	Total	ng/mL	10	6.3	6.0	11

a Number of observations.

**Table 6:** Weighted percent measurable estimates.

			As form, percent measurable							
Study	Media	No. of samples speciated	As(V)	As(III)	DMA	MMA	AsB	AsC	Total	No. of total samples
R5	Food	159	2.2	0.0	0.4	0.0	12.9	0.0	99.7	159
CS	Food	101	3.3	0.0	0.0	0.0	15.2	0.0	100.0	99
	Dust		—	—	—	—	—	—	99.4	101
	Hair		—	—	—	—	—	—	47.0	79
	Water	85	78.0	4.7	2.7	0.8	—	—	99.7	102
	Urine, day 3	82	1.7	1.3	70.5	9.9	14.4	0.0	100.0	79
	Urine, day 5	86	17.0	2.7	65.2	17.2	17.6	0.0	100.0	83
	Urine, day 7	83	8.7	1.4	72.8	18.0	19.7	1.8	100.0	83

—, no data.

**Table 7:** Population-weighted estimates.^a^

													95% confidence limits					
Study	Media	As Form	Units	No.^b^	Population size (1,000s)	Percent meas.	Mean	10th	25th	Median	75th	90th	Mean	10th	25th	Median	75th	90th
R5																		
	Food	Total	μg/kg	159	47,548	99.7	17.18	3.57	4.87	7.72	17.74	43.05	12.35^c^	2.74	3.97	6.06	12.08	23.66
													22.01^d^	4.88	6.25	9.86	25.30	46.36
	Food	Total	μg/day	158	47,403	99.7	12.67	1.65	2.53	5.04	13.82	31.65	8.63	1.02	2.09	3.52	8.03	18.86
													16.72	2.20	3.51	7.58	19.57	44.81
CS																		
	Food	Total	μg/kg	99	85	100.0	32.41	9.79	13.09	17.50	25.99	46.58	13.50	8.57	11.33	15.05	19.90	28.50
													51.33	11.62	15.15	20.17	34.15	92.25
	Food	Total	μg/day	99	85	100.0	16.46	4.23	5.46	8.71	13.33	28.58	6.79	3.57	5.08	7.58	10.50	15.06
													26.14	5.29	7.59	10.93	19.91	44.88
	Dust	Total	ng/cm^2^	101	48	99.4	0.41	0.03	0.07	0.16	0.45	1.32	0.23	0.03	0.05	0.11	0.27	0.49
													0.60	0.06	0.11	0.30	0.84	1.65
	Dust	Total	μg/g	101	48	99.4	9.41	3.13	3.45	7.40	11.25	20.59	7.52	2.24	3.27	5.31	8.56	11.81
													11.31	3.44	6.32	8.92	12.70	25.98
	Hair	Total	μg/g	79	88	47.0	0.17	0.06	0.08	0.12	0.23	0.33	0.14	0.05	0.07	0.09	0.19	0.24
													0.20	0.08	0.10	0.20	0.28	0.38
	DW	As(V)	ng/mL	85	47	78.0	0.47	—	0.25	0.38	0.60	0.88	0.37	—	—	0.33	0.42	0.61
													0.58	0.25	0.34	0.51	0.83	1.07
	DW	Total	ng/mL	102	49	99.7	0.88	0.51	0.61	0.80	1.01	1.31	0.78	0.48	0.53	0.75	0.84	1.13
													0.98	0.55	0.75	0.84	1.19	1.50
	Urine,	DMA	ng/mL	82	83	70.5	5.48	—	—	3.91	6.54	11.44	3.74	—	—	2.80	4.89	7.18
	day 3												7.22	—	3.56	4.95	8.56	—
		Total	ng/mL	79	83	100.0	14.38	1.68	4.95	9.79	14.58	36.10	9.58	1.10	2.77	7.44	11.34	17.04
													19.19	4.80	8.39	12.30	25.95	—
	Urine,	DMA	ng/mL	86	82	65.2	5.77	—	—	4.22	6.31	9.13	2.81				4.98	6.46
	day 5												8.74	—	3.35	5.41	9.04	10.42
		Total	ng/mL	83	79	100.0	16.37	2.02	4.43	10.23	17.13	25.97	7.90	1.44	2.80	8.43	12.83	17.15
													24.84	4.17	9.17	13.41	20.31	—
	Urine,	DMA	ng/mL	83	80	72.8	6.39	—	—	4.33	7.30	9.78	3.10	—	—	3.25	5.73	8.23
	day 7												9.68	—	3.39	5.94	8.93	—
		Total	ng/mL	83	80	100.0	49.48	1.22	5.85	10.77	17.02	25.57	−23.33	1.07	2.22	8.33	14.20	17.83
													122.28	5.50	9.23	15.14	20.62	—

meas., measurable. 10th, 25th, 75th, and 90th are percentiles.

a 0.5 times the detection limit was substituted for nondetectable values.

b Number of observations.

c Low confidence limit.

d High confidence limit.

**Table 8:** Spearman correlations of As and As species: biomarkers versus environmental and exposure measures.

	Food					
Biomarker concentration	AsB (μg/kg)	Total (μg/kg)	AsB (μg/day)	Total (μg/day)	Water AsV (μg/L)	Dust Total (μg/g)
Urine, day 3, Total	0.266	−0.012	0.299*	0.170	0.213	−0.178
Urine, day 5, Total	0.065	0.280**	0.001	0.167	0.192	0.065
Urine, day 7, Total	0.159	0.276**	0.280**	0.318*	0.122	−0.219**
Urine, day 3, As(V)	−0.087	−0.012	−0.027	−0.065	0.193	−0.052
Urine, day 5, As(V)	−0.092	0.091	−0.052	0.058	−0.173	0.182
Urine, day 7, As(V)	0.190	0.118	0.178	0.123	−0.007	−0.034
Urine, day 3, AsB	0.145	−0.039	0.228**	0.112	0.212	0.023
Urine, day 5, AsB	0.284*	0.255**	0.228 **	0.241**	−0.053	−0.052
Urine, day 7, AsB	0.485*	0.331*	0.425 *	0.370*	0.006	−0.162
Urine, day 3, DMA	0.052	−0.031	0.156	0.064	0.305**	−0.107
Urine, day 5, DMA	0.069	0.203	−0.038	0.127	0.123	0.068
Urine, day 7, DMA	0.113	0.169	0.093	0.153	−0.050	−0.154
Urine, day 3, MMA	0.169	−0.058	0.055	−0.067	0.299**	−0.129
Urine, day 5, MMA	0.043	0.109	−0.008	0.084	0.040	0.117
Urine, day 7, MMA	0.062	0.165	−0.033	0.043	0.197	−0.149
Hair, total	−0.003	0.089	0.050	0.049	0.028	0.059

* Statistically significant at the 0.01 level.

** Statistically significant at the 0.05 level.

**Table 9:** Pearson log-scale correlations of As and As species: biomarkers versus environmental and exposure measures.

	Food				Water		Dust		
Biomarker concentration	AsB (μg/kg)	Total (μg/kg)	AsB (μg/day)	Total (μg/day)	As(V) (μg/L)	Total (μg/L)	Total (μg/cm^2^)	Total (μg/g)	Hair Total (μg/g)
Urine, day 3, Total	0.206	0.124	0.273	0.217	0.186	0.017	−0.148	−0.175	−0.197
Urine, day 5, Total	0.314*	0.365*	0.250**	0.280**	0.179	0.220**	−0.080	−0.059	−0.027
Urine, day 7, Total	0.510*	0.465*	0.520*	0.487*	0.126	0.113	−0.223**	−0.194	−0.013
Urine, day 3, As(V)	−0.080	−0.057	−0.088	−0.070	0.354*	0.282**	−0.044	−0.047	0.236
Urine, day 5, As(V)	−0.025	0.012	−0.040	−0.010	−0.095	−0.044	0.075	0.114	0.139
Urine, day 7, As(V)	0.411*	0.326*	0.408*	0.331*	0.017	−0.073	−0.071	−0.009	−0.097
Urine, day 3, AsB	0.005	−0.041	0.091	0.077	0.200	−0.003	0.039	0.061	−0.017
Urine, day 5, AsB	0.512*	0.437*	0.499*	0.428*	−0.015	−0.025	−0.038	−0.057	−0.092
Urine, day 7, AsB	0.803*	0.677*	0.761*	0.635*	0.018	0.046	−0.148	−0.074	−0.029
Urine, day 3, DMA	0.040	0.027	0.098	0.104	0.248	−0.067	−0.144	−0.087	−0.236
Urine, day 5, DMA	0.223**	0.284*	0.187	0.233**	0.115	0.184	0.027	0.065	0.096
Urine, day 7, DMA	0.363*	0.319*	0.343*	0.301*	−0.019	0.033	−0.138	−0.105	0.119
Urine, day 3, MMA	0.118	0.061	0.119	0.065	0.291**	0.012	−0.108	−0.119	−0.119
Urine, day 5, MMA	0.156	0.185	0.141	0.165	0.031	−0.023	0.038	0.104	0.089
Urine, day 7, MMA	0.110	0.143	0.064	0.080	0.115	−0.042	−0.100	−0.204	0.102
Hair total	−0.000	0.013	0.016	0.037	0.095	0.166	0.158	0.048	

* Statistically significant at the 0.01 level.

** Statistically significant at the 0.05 level.

**Table 10:** Spearman and log-scale Pearson correlations between urine samples.

	Spearman			Pearson (log-scale)		
As form	Day 3 vs. day 5	Day 5 vs. day 7	Day 3 vs. day 7	Day 3 vs. day 5	Day 5 vs. day 7	Day 3 vs. day 7
Total	0.245**	0.269**	0.412*	0.311*	0.449*	0.472*
As(V)	0.060	0.052	−0.074	−0.061	0.096	−0.069
AsB	0.390*	0.443*	0.403*	0.262**	0.521*	0.280**
DMA	0.191	0.238**	0.216	0.225**	0.305*	0.249**
MMA	0.094	0.300*	0.199	0.078	0.317*	0.143

* Statistically significant at the 0.01 level.

** Statistically significant at the 0.05 level.
